# Automated assignment grading with large language models: insights from a bioinformatics course

**DOI:** 10.1093/bioinformatics/btaf196

**Published:** 2025-07-15

**Authors:** Pavlin G Poličar, Martin Špendl, Tomaž Curk, Blaž Zupan

**Affiliations:** Faculty of Computer and Information Science, University of Ljubljana, Večna pot 113, 1000 Ljubljana, Slovenia; Faculty of Computer and Information Science, University of Ljubljana, Večna pot 113, 1000 Ljubljana, Slovenia; Faculty of Computer and Information Science, University of Ljubljana, Večna pot 113, 1000 Ljubljana, Slovenia; Faculty of Computer and Information Science, University of Ljubljana, Večna pot 113, 1000 Ljubljana, Slovenia; Department of Education, Innovation and Technology, Baylor College of Medicine, 1 Baylor Plz, Houston, TX 77030, United States

## Abstract

**Motivation:**

Providing students with individualized feedback through assignments is a cornerstone of education that supports their learning and development. Studies have shown that timely, high-quality feedback plays a critical role in improving learning outcomes. However, providing personalized feedback on a large scale in classes with large numbers of students is often impractical due to the significant time and effort required. Recent advances in natural language processing and large language models (LLMs) offer a promising solution by enabling the efficient delivery of personalized feedback. These technologies can reduce the workload of course staff while improving student satisfaction and learning outcomes. Their successful implementation, however, requires thorough evaluation and validation in real classrooms.

**Results:**

We present the results of a practical evaluation of LLM-based graders for written assignments in the 2024/25 iteration of the Introduction to Bioinformatics course at the University of Ljubljana. Over the course of the semester, more than 100 students answered 36 text-based questions, most of which were automatically graded using LLMs. In a blind study, students received feedback from both LLMs and human teaching assistants (TAs) without knowing the source, and later rated the quality of the feedback. We conducted a systematic evaluation of six commercial and open-source LLMs and compared their grading performance with human TAs. Our results show that with well-designed prompts, LLMs can achieve grading accuracy and feedback quality comparable to human graders. Our results also suggest that open-source LLMs perform as well as commercial LLMs, allowing schools to implement their own grading systems while maintaining privacy.

## 1 Introduction

The recent development and widespread availability of large language models (LLMs) have led to their adoption across numerous fields of human endeavor (Kaddour *et al.* 2023, Minaee *et al.* 2024). Their ability to provide instant and personalized responses has naturally prompted researchers to explore their use in education, revealing applications that benefit both students and instructors. These applications take various forms, including personalized student tutoring (Lyu *et al.* 2024), contextualizing exercises to enhance engagement (Yadav *et al.* 2023), and automated grading of student submissions (Chiang and Lee 2023, Liu *et al.* 2023).

In addition to reducing the workload on teaching faculty, automated grading offers numerous benefits to students and their educational outcomes. Studies have shown that students prefer feedback that is both linguistically clear and provided in a timely manner (Paterson *et al.* 2020). Encouraging and constructive feedback has also been linked to improved academic performance. Furthermore, automated grading ensures greater consistency in scoring and feedback, as LLMs are not prone to human errors such as fatigue and variability in grading standards (Klein 2002, Madigan *et al.* 2023). This approach allows teaching assistants (TAs) to dedicate more time to direct interactions with students, which students also highly value (Paterson *et al.* 2020).

Automatic grading of student assignments dates back to as early as 1968 (Page 1968). Since then, several systems for grading short answers have been developed, typically relying on a corpus of annotated responses (Mohler *et al.* 2011, Riordan *et al.* 2017). However, the emergence of LLMs with few-shot capabilities makes them particularly well suited for automated grading, especially in cases where instructors can anticipate correct answers and common mistakes. As a result, adopting this technology has become more feasible than ever.

Several studies have explored the use of LLMs in the classroom. Kostic *et al.* (2024) examined GPT-4’s ability to grade essays and reported poor performance. They also investigated grading variability among human instructors in a small workshop setting; however, their study was limited to only three instructors grading four essays. Similarly, Dai *et al.* (2023) used ChatGPT to generate feedback for student project proposals and found that, while ChatGPT was consistently able to generate more readable and clearer feedback than human instructors, its assessment performance proved to be inadequate for a real-classroom setting. In contrast, Impey *et al.* (2024) applied GPT-4 to grade submissions from three massive open online courses and found assessment performance comparable to that of instructors and outperforming peer-based grading. However, their study focuses primarily on assessment performance and largely overlooks the importance of providing constructive feedback. While the aforementioned studies investigated LLM-based grading retrospectively, Chiang *et al.* (2024) integrated GPT-4 into a real-world course, “Introduction to Generative AI.” Students had direct access to GPT-4 and the associated grading prompts (i.e. prompts for grading) and were allowed to test their responses up to 80 times per assignment. In their study, students’ final grades were determined by the scores they were able to achieve using the LLM.

**Figure 1.**
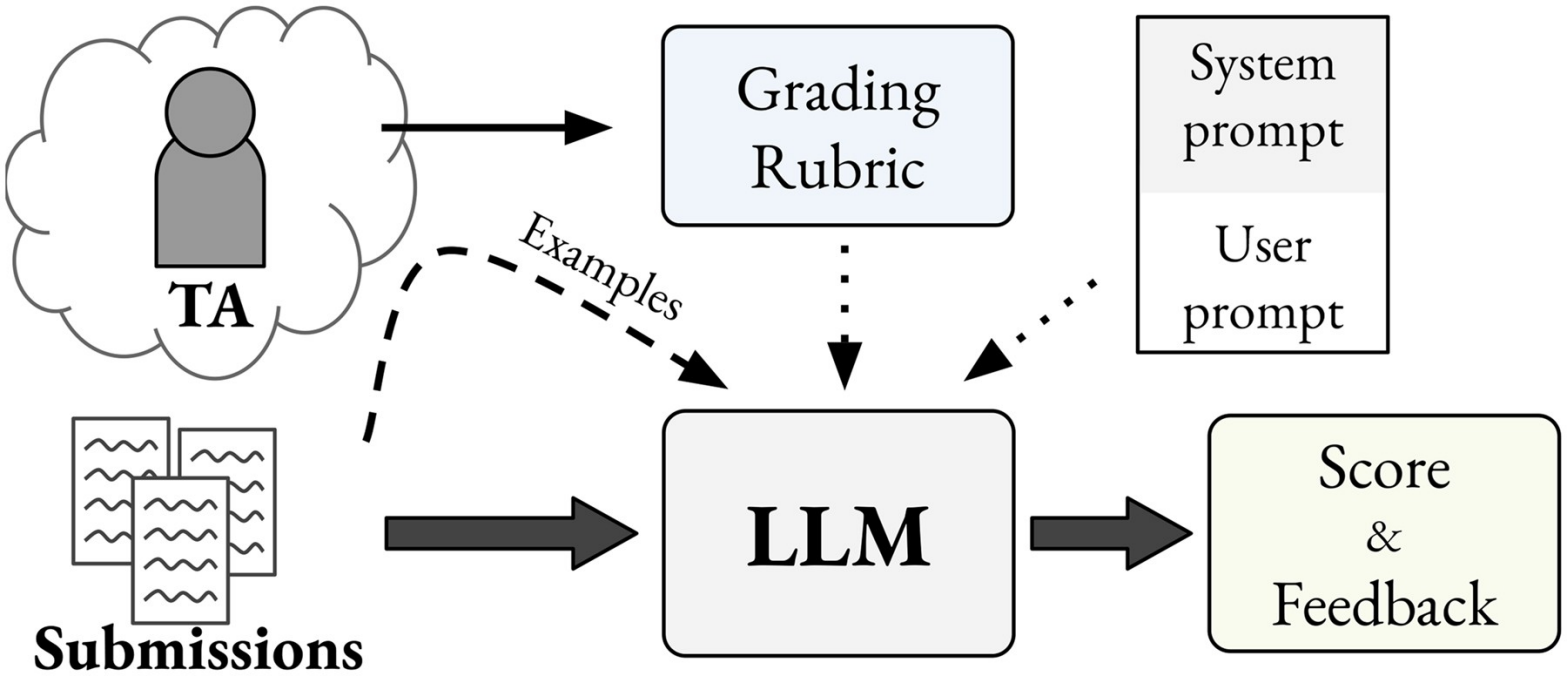
Schema of student submissions graded by LLMs, based on TA-graded examples and grading rubric composed of criteria. System and user prompts serve as standard role-based instructions for LLMs. The dashed arrow denotes 25% of students’ submissions that were graded by TAs for in-context examples.

In this study, we examine the use of LLM graders in a university classroom setting applied to the Introduction to Bioinformatics course, a hands-on bioinformatics course whose innovative design and focus on practical problems we previously reported at ISMB-24 (Poličar *et al.* 2024). Unlike Chiang *et al.* (2024), where students had access to LLM-generated grading prompts, we used LLMs as direct replacements for human graders, grading student submissions only once after the assignment due date, without providing students access to the grading prompts (see Fig. 1). This setup closely reflects real-world grading scenarios and serves as a valuable case study for implementing LLMs in other academic settings. Additionally, the study was conducted in a randomized manner, where students were unaware of whether their submissions were graded by a human or an LLM. Students subsequently evaluated the quality of the feedback they received, enabling a quantitative comparison between human and machine grading. While most existing studies focus on a single LLM, typically GPT-4, we systematically compare the performance of six different LLMs as automated graders and benchmark them against human TAs.

The study design was reviewed and approved by our institutional internal review board—the Research Ethics and Data Handling Review Board of the University of Ljubljana (approval number 20241130001)—to ensure compliance with ethical research standards.

## 2 Study design

We conducted our study in the introductory course to bioinformatics offered by the Faculty of Computer and Information Science, University of Ljubljana, during the 2024–25 winter semester. The course is taught in English. This year’s cohort included 119 students, primarily master’s level computer science students, but also included several students from the Faculty of Mathematics and Physics and the Biotechnical Faculty. The course comprises lectures, five take-home assignments, and a final exam. Each of the five take-home assignments tackles a different aspect of bioinformatics, following the SARS-CoV-2 case study detailed in our previous work (Poličar *et al.* 2024). Each assignment consists of multiple exercises in which students implement bioinformatics algorithms, apply them to real-world data, visualize their findings, and discuss their results in written answers to specific questions. Each assignment contains several mandatory exercises designed to guide students through an investigation of the SARS-CoV-2 virus. Students can earn extra points by completing bonus exercises that complement the main storyline. After each assignment deadline, the TAs assess each student’s submission and provide a numeric score for the overall assignment, as well as written feedback clarifying mistakes and offering potential improvements. In our standard execution of the course, programming exercises are graded using automatic unit tests that verify the correctness of the algorithm implementations, while figure submissions and text answers are graded manually by the TA.

In the present study, we investigate whether LLMs could be used in place of human TAs for the assessment of written text answers. Participating students had their text-based answers reviewed and graded by an LLM. Unless the student requested a human review of the grade, the LLM-assigned grades were used in their final grades. Consent was obtained for each of the five assignments. Participation was purely voluntary, and a student’s decision on whether or not to participate had no bearing on the student’s final grades. Students withholding their consent had their assignments graded in our standard manner, using automated unit tests and human review. Study participation rates were high. On average, we received 105 submissions for each of the five assignments, where between 99 and 101 (∼94%) students gave consent to be included in the study. Overall, 93 students gave consent for all five assignments.

The study was performed as follows. Each of the five assignments includes between 2 and 7 mandatory essay-style questions and between 1 and 3 optional bonus essay-style questions. Each textual response was randomly assigned to one of the eight groups—two TA-based and six LLM-based—where either a TA or LLM assigned a score and provided written feedback according to the same predefined grading rubrics. This feedback was interspersed with unit test-generated feedback from programming exercises and TA-written feedback for figure submissions. Consequently, students receive grades and feedback from multiple graders on textual questions in a single assignment. The students were not informed which grader evaluated each of their text-based answers and did not have access to the prompts at any point. Upon receiving their assignment grade and feedback, we ask students to fill out a survey rating their satisfaction with the feedback on each of the text-based questions in their assignment. Due to the potential for LLM errors, participating students may request a human review of any of the answers. If no reevaluations are requested, the LLM-assigned grades are used as their final grades. We note that the TAs were informed that their grades and feedback would be compared to that of LLMs as part of this study. While this awareness may have led them to be more careful in their assessments, this additional scrutiny likely improved the quality and reliability of the reference grading.

The study aimed to compare popular commercial and noncommercial LLMs. To assess the capabilities of LLMs for grading student-written text submissions, we include three different LLM model architectures, including the popular ChatGPT model (GPT-4o) from OpenAI (OpenAI 2024), four different versions of the open-source Llama 3 models from Facebook (AI@Meta 2024), and a recent model from NVIDIA (Llama-3.1-Nemotron-70B, referred to as Nvidia-70B) (Wang *et al.* 2025). Facebook released three open-source versions of the Llama 3 architecture with varying numbers of parameters: 7B, 70B, and 405B. While the larger of these models require specialized hardware, which is often not available to university departments, the smaller models can be run on high-end consumer-grade GPUs, which can more readily be found in university departments. Additionally, the hardware requirements can often be reduced through quantization, often at minimal loss in performance (Jin *et al.* 2024). In our study, we include full-precision versions of Llama-8B and Llama-70B, as well as quantized versions of Llama-70B and Llama-405B, which we denote as Llama-70Bq4 and Llama-405Bq4, respectively. The full-precision version of Llama-405 was not included due to hardware limitations, while a quantized version of Llama-8B was not included based on poor performance in preliminary preparations for this study. To preserve sufficient statistical power in comparisons among LLMs, we did not consider other available models. In total, we include six LLMs: GPT-4o, Nvidia-70B, Llama-405Bq4, Llama-70B, Llama-70Bq4, and Llama-8B.

A key requirement for an effective LLM grader is the ability to provide high-quality feedback. As described earlier, we assess feedback quality through student surveys completed after receiving their graded assignments. However, there are multiple aspects that humans take into account when evaluating written feedback, of which we identify tone and content as the two most important aspects. To disentangle the impact of tone from content in student preferences, we include an additional grading group: “TA-GPT-revised.” In this group, human TAs assign scores and provide written feedback, which is then rewritten by GPT-4o-mini. The model is instructed to preserve the original content while adjusting only the tone to match ChatGPT’s typical style. By comparing student satisfaction between these two groups, we are able to discern whether student preferences are driven by differences in tone, content, or both.

## 3 Prompts

**Figure 2.**
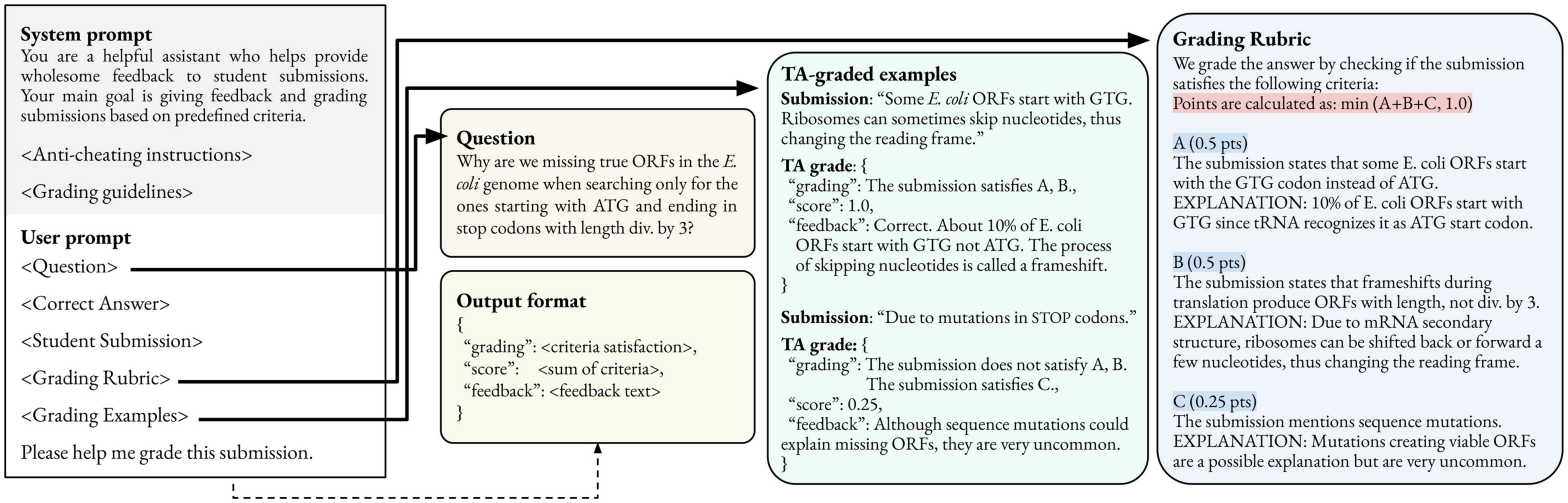
Prompt structure with a grading rubric and TA-graded examples. The system prompt is fixed across exercises, while the user prompt dynamically includes the associated question, examples of correct answers, grading rubrics, and graded examples. The model response is structured as a JSON with predefined fields.

Each student’s answer is evaluated using a single prompt for an LLM comprised of a fixed system prompt and an exercise-specific user prompt. The system prompt includes general grading instructions and guidelines, while the user prompt includes exercise-specific information, including the question, a sample correct answer, the student submission, the grading rubric, and several TA-graded examples. The overall prompt structure is shown in Fig. 2. The user prompts consist of two key components: the grading rubric, which specifies the grading criteria and corresponding point allotments, and manually graded grading examples of student submissions for the specific exercise. We describe each of these in more detail below.

Each grading rubric consists of one or more grading criteria, each specifying a required aspect or theme that must be present in the submission to earn points. Each criterion is allotted a certain number of points, and the total score is computed by summing the points from all satisfied criteria. Criteria can also include an optional explanation section that can be used solely for feedback. This allows LLMs to draw on additional information when generating written feedback, which may be helpful in certain explanations, such as a particularly illustrative or interesting example from biology that students are not expected to know or a simple counterexample demonstrating why a particular answer was incorrect. In some instances, satisfying all criteria would result in a score exceeding 100%. In these cases, we include a formula that specifies the exact computation of the final score (highlighted in red in Fig. 2).

The blue panel in Fig. 2 shows one particular grading rubric comprised of three grading criteria. Each criterion is accompanied by an explanation. In this example, criteria A and B denote both parts of the correct answer (0.5 points each), but partial points can also be achieved via criteria C (0.25 points). Since a comprehensive student answer could satisfy all three criteria, simply adding the points together would yield a score of 1.25 points. Therefore, we include an expression in the preamble of the rubric table specifying how the final score should be obtained (see Fig. 2, red). In the present study, we consider only additive criteria, as this simplifies grading rubric design and enables more transparent grading and feedback. However, we have no reason to believe subtractive criteria would perform differently.

The grading examples section contains up to 10 examples of manually graded submissions per exercise. To ensure a diverse set of graded examples, the manually graded submissions from the TA and TA-GPT-corrected grading groups are first grouped based on unique combinations of satisfied grading criteria (e.g. *satisfies A but not B*). Then, both groups and submissions are sampled randomly to be included in the prompt. The green panel in Fig. 2 shows two examples of graded examples.

As shown in Fig. 2, LLMs are prompted to return a structured response containing the score and written feedback for each submission, as well as a list of satisfied rubric criteria. During informal preliminary testing of different prompts, we found that requiring LLMs to explicitly list the satisfied rubric criteria improves assessment accuracy. While we could programmatically parse the list of satisfied criteria and compute the total score of each submission, we have found that LLMs reliably handle this task and that mistakes are extremely rare. Apart from a single instance (0.3%) of undercounting by LLama-8B out of its 333 submissions, no other model made errors in tallying points.

### 3.1 Preparing grading rubrics

Next, we describe our approach to preparing structured grading rubrics compatible with LLMs. Based on initial discussions among the TAs and course instructors, we first prepare preliminary grading rubrics for each of the 36 questions, specifying correct and partially correct answers. Each grading rubric comprises between 1 and 4 grading criteria, resulting in a total of 61 grading criteria across all questions. We then manually correct a sample of student submissions and make adjustments to the rubrics as needed. As part of this study, 25% of the text-based submissions are assigned to the TA or TA-GPT-revised grading groups. We use these submissions to assess and refine the grading rubrics. To verify that the grading rubric is compatible with LLMs, we evaluate these submissions using GPT-4o and manually inspect any mismatches between TA-assigned and LLM-assigned scores. In case of systematic differences in the LLM-assigned scores due to, e.g., a poorly worded prompt, we revise the grading rubric as needed. We then use the revised grading rubrics to evaluate these same submissions again and inspect whether the identified errors were resolved.

In practice, major changes to the grading rubric were rare, and anecdotally, most revisions involved rewording and clarifying ambiguous criteria. Although the procedure outlined above applies preferential treatment to the GPT-4o model, our intent here is not to tailor prompts to any particular model but rather to identify systematic problems with our prompts. To minimize the risk of overfitting to any one particular model, we limit ourselves to a single round of prompt refinement. We have found that this is often enough to identify and correct the most systematic errors. As we will later see in the Section 4, despite this advantage, GPT-4o performs comparably to other similarly sized open-source models, indicating minimal overfitting.

## 4 Results

Here, we consider two aspects of grading, both of which inform students about their performance: the numeric score assigned to each exercise and the accompanying written feedback. We aim to answer the following two questions: (i) do LLMs provide accurate grades? and (ii) is the feedback they generate useful? In order for LLMs to serve as viable replacements for human TAs, they must perform well on both tasks.

### 4.1 LLM grading accuracy

Student submissions assigned to the TA and TA-GPT-revised served as the ground truth for evaluating LLM performance. This subset accounts for 25% of total submissions, comprising 670 manually graded submissions across 36 text-based exercises. Each submission was assigned a score between 0 and 1, following the grading rubrics outlined in Section 3. These grades were then used as the gold standard against which we compare the performance of different LLMs. To decompose the performance of LLMs across different exercise difficulty levels, the TAs manually categorized each of the 36 exercises into five difficulty categories: “trivial” (*n* = 5, $\mu_{\text{score}}=0.96$, 95% CI [0.92, 0.99]), “easy” (*n* = 14, $\mu_{\text{score}}=0.92$, 95% CI [0.89, 0.95]), “medium” (*n* = 11, $\mu_{\text{score}}=0.81$, 95% CI [0.75, 0.86]), “hard” (*n* = 4, $\mu_{\text{score}}=0.40$, 95% CI [0.31, 0.49]), and “open-ended” (*n* = 2, $\mu_{\text{score}}=0.90$, 95% CI [0.83, 0.96]). The reported mean scores $\mu_{\text{score}}$ suggest that the ranking they devised was consistent with student performance within each exercise difficulty category.

At first glance, a direct evaluation of LLM graders would compare the number of points assigned by LLMs to those assigned by human TAs. However, in our particular submission scoring setup—where points are awarded based on correctly identifying satisfied grading criteria—directly comparing the number of points would not provide an accurate assessment of LLM performance. For exercises with a single grading criterion, a perfect score depends on correctly judging a single criterion. For exercises with multiple criteria, however, LLMs must make several correct judgments in order to award a perfect score, increasing the chance of errors. Thus, directly comparing numeric scores biases evaluation performance in favor of exercises with a single grading criterion. Consequently, framing this task as a binary classification problem, in which LLMs judge whether a particular criterion was satisfied or not, provides a more reliable measure of model performance. While many different metrics are available for assessing binary classification performance, we here report the classification accuracy (CA), which measures the proportion of correct judgments made and allows us to easily identify LLM grading biases in terms of leniency (awarding more points than TAs) and strictness (awarding less points than TAs).

**Figure 3.**
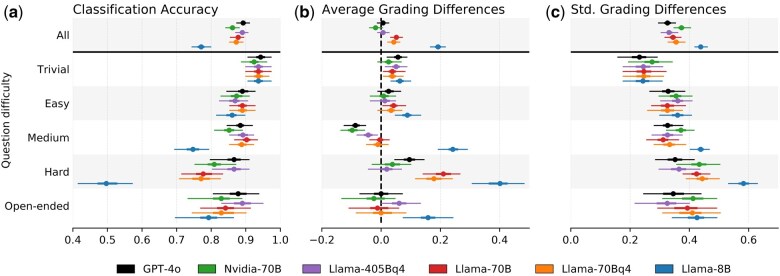
LLM performance on predicting grading criteria. TA grades represent the gold standard. 95% confidence intervals (CI) of summary statistics are obtained using bootstrap samples. (a) Classification accuracy of LLMs predicting each satisfied criteria as a binary classification. (b) The average grading difference in prediction indicates more lenient (positive) or stringent (negative) grading by the LLM compared to TAs. (c) The standard deviation of the grading difference indicates consistency among models.

The top panel of Fig. 3a shows the overall CA of each of the LLMs. Overall, LLMs achieve strong performance, with average CA scores ranging between 85% and 90%. One notable exception is Llama-8B, which achieves a relatively poor CA of 75%. When grouping exercises by difficulty, we notice a decrease in CA as the difficulty of the exercises increases. This is likely because harder-to-answer questions often receive wildly varying answers that are impossible to foresee and define their scoring within the prompts. Hard questions, in particular, often require longer answers that sometimes contain mathematical equations, which may be difficult for models to categorize appropriately. One particularly interesting category of questions is open-ended questions, where there is no one particular correct answer. These kinds of questions pose an interesting challenge. For open-ended questions, it is often impractical to exhaustively list all possible correct, and the final judgment must often be made by the LLM. Despite this, LLMs generally achieve solid performance, achieving accuracies between 80% and 90%. One interesting observation here is that in the “hard” and “open-ended” categories, model performance appears to closely match the number of model parameters. Both GPT-4o and Llama-405Bq4 achieve similar performance, while the 70B models all achieve slightly lower performance. Llama-8B performs substantially worse still.

While the CA reports on the proportion of correct judgments, it does not reveal whether models tend to be more lenient or stringent in grading than TAs. In Fig. 3b, we plot the average differences in the matched grading criteria. Positive values indicate that LLMs were more lenient, classifying more criteria as satisfied than TAs. Negative values indicate that models failed to report many of the criteria that TAs marked as satisfied, resulting in lower final grades. For trivial and easy questions, models exhibit little systematic bias with means differences close to zero. For medium-difficulty questions, the models show low levels of negative bias, while the opposite is true for difficult questions, where most models are significantly more lenient than TAs. In open-ended questions, the majority of models exhibited no systematic bias. Finally, we show the variance of differences in Fig. 3c, which shows minimal differences between models.

The obvious exception to the above is Llama-8B, which exhibits poor performance across the board and is overly lenient in its grading. For instance, on hard questions, Llama-8B correctly graded only about 50% of submissions and assigned too many points in about 45% of submissions and too few points in about 5% of submissions. The poor performance of Llama-8B indicates its unsuitability for its use as an assignment grader in the classroom. Its poor performance could be due to several factors. Firstly, our prompts are quite long, and perhaps Llama-8B struggles with the context size. Interestingly, however, this does not appear to be the case with easier questions, making this explanation unlikely. Secondly, we designed our prompts to be generic and not tailored to any one specific model in particular. It is plausible that the current format is not compatible with Llama-8B and that we might achieve better performance if prompts were specifically tailored to Llama-8B. However, given that the remaining models did not appear to require such adaptations, we anticipate that the most likely explanation is due to a final explanation—the inherent limitations of smaller models. This is supported by the fact that Llama-8B appears to have the most difficulties with harder questions, which typically require longer, more involved answers that include, for instance, several steps of reasoning or short mathematical proofs for which Llama-8B perhaps lacks the reasoning capabilities to fully understand. On the other hand, 4-bit quantized Llama-70Bq4 with similar hardware requirements performed much better and achieved near nonquantized performance.

From the analysis above, we make the following observations:

1. With the exception of Llama-8B, all models achieve adequate performance, demonstrating high accuracy when determining whether a particular submission satisfies predefined grading criteria and exhibits low levels of systematic bias.
2. Model performance generally correlates with their number of parameters. The larger GPT-4o and Llama-405Bq4 models perform favorably to the 70B parameter models, which in turn outperform the 8B parameter Llama variant.
3. Quantization appears to have a negligible effect on performance, as the quantized variant of the Llama-70B model achieves comparable performance to its full-precision counterpart.
4. Although none of the models achieve perfect accuracy, we have determined their margin of error to be acceptable. Given the general direction of the grading biases, we anticipate little student pushback. Furthermore, students who suspect grading errors can request a manual review.

### 4.2 Impact of including grading rubric and grading examples

**Figure 4.**
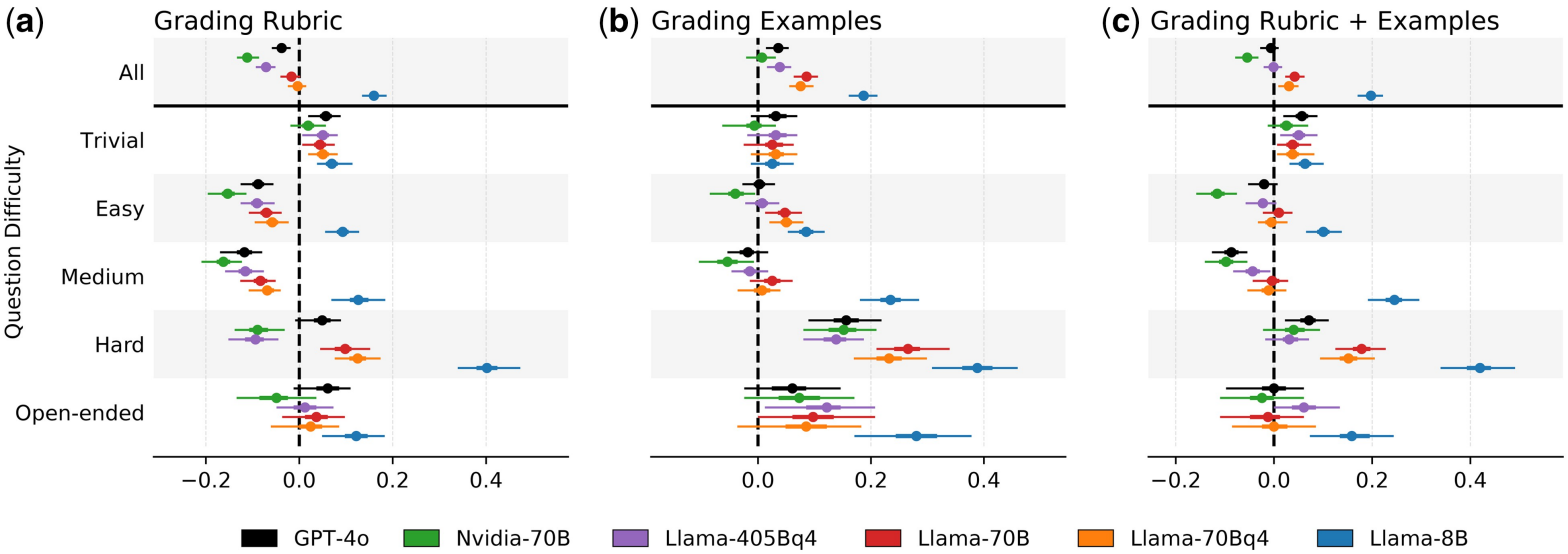
The importance of including grading rubrics and graded examples on LLM performance. The scale relates to systematic bias with respect to TA grades. 95% confidence intervals (CI) of summary statistics are obtained using bootstrap samples. (a) LLM performance using only the TA-defined grading rubric in the user prompt. (b) LLM performance using only TA-graded examples without the grading rubric. (c) LLM performance using both a grading rubric and graded examples in the user prompt. Since we include both grading rubrics and grading examples in our final grading prompts, this panel is the same as Fig. 3b.

In the previous section, we examined the performance of different LLMs using prompts that included both grading rubrics and grading examples. Here, we investigate the effects of excluding each of these elements from the prompt. While these variants were not used in the actual submission assessment, our results highlight the importance and effects of each component. In Fig. 4, we report the mean differences in the matched grading criteria for the three different prompt variants.

The top rows of Fig. 4 show the performance of the six LLMs across the three prompt variants. Prompts that included only grading rubrics led to stricter grading, with LLMs less likely to match grading criteria (Fig. 4a). On the other hand, prompts that included only grading examples resulted in more lenient grading, as LLMs were overly generous (Fig. 4b). Including both the grading rubric and grading examples produced the best results, achieving a middle ground between the two individual results. Curiously, these biases did not greatly affect their CA, which was predominantly not statistically significant; all three variants achieved similar CA scores (not shown for brevity).

We might expect that given enough examples, LLMs could infer the grading rubric internally, potentially eliminating the need for course instructors and TAs to prepare detailed grading rubrics. The results from Fig. 4b indicate that, for simpler questions, LLMs achieve satisfactory performance using grading examples alone. However, for harder and open-ended questions, we observe a marked drop in performance. We hypothesize that this may be due to the increased variability in student responses. Simpler questions tend to have more straightforward answers with limited variation. Since we include several manually graded student submissions in the grading prompt, most student responses will likely be similar to the grading examples, giving LLMs a blueprint for the desired response. In contrast, answers to more difficult and open-ended questions are often longer and more varied, making it less likely that the grading examples will cover the wide range of possible answers. For these more difficult questions, providing a grading rubric is essential (see Fig. 4c).

### 4.3 Student preferences for LLM-based feedback

Feedback is a fundamental aspect of the learning process, and effective feedback has been shown to improve learning outcomes (Paterson *et al.* 2020). Upon receiving grades and feedback for each of the five assignments, we asked students to rate the feedback received for each text-based answer after receiving feedback for each assignment. We received student satisfaction scores for a total of 1527 answers, of which 1189 were correct, and 338 were incorrect or partially correct.

To determine whether students prefer human-written or LLM-generated written feedback, we model the student satisfaction score using a Bayesian mixed-effects ordered probit regression (Kruschke 2015):


(1) $$\begin{array}{c} \mu_{i}=\gamma_{m_{i}}+\eta_{e_{i}}+\psi_{s_{i}}+\alpha \cdot {\text{score}}_{i}+\tau \cdot {\text{total}}_{i},\\ y_{i}∼\text{OrderedProbit}(\mu_{i},\text{cutpoints}), \end{array}$$

where $y_{i}$ denotes the student satisfaction score for a particular text-answer *i*. Here, $\gamma_{m_{i}}$ corresponds to the grading group factor (one for each of the eight groups), $\eta_{e_{i}}$ represents the factor assigned to each exercise, accounting for different difficulty levels of the exercises, and $\psi_{s_{i}}$ accounts for individual student biases. Since higher exercise and assignment scores typically lead to higher satisfaction ratings, we model these effects explicitly using the coefficients α (scores of individual exercises) and τ (score of the entire assignment). We assign uninformative priors N(0,2) on all parameters and perform inference using the Stan library using Hamiltonian Monte Carlo sampling (HMC) (Stan Development Team 2025).

**Figure 5.**
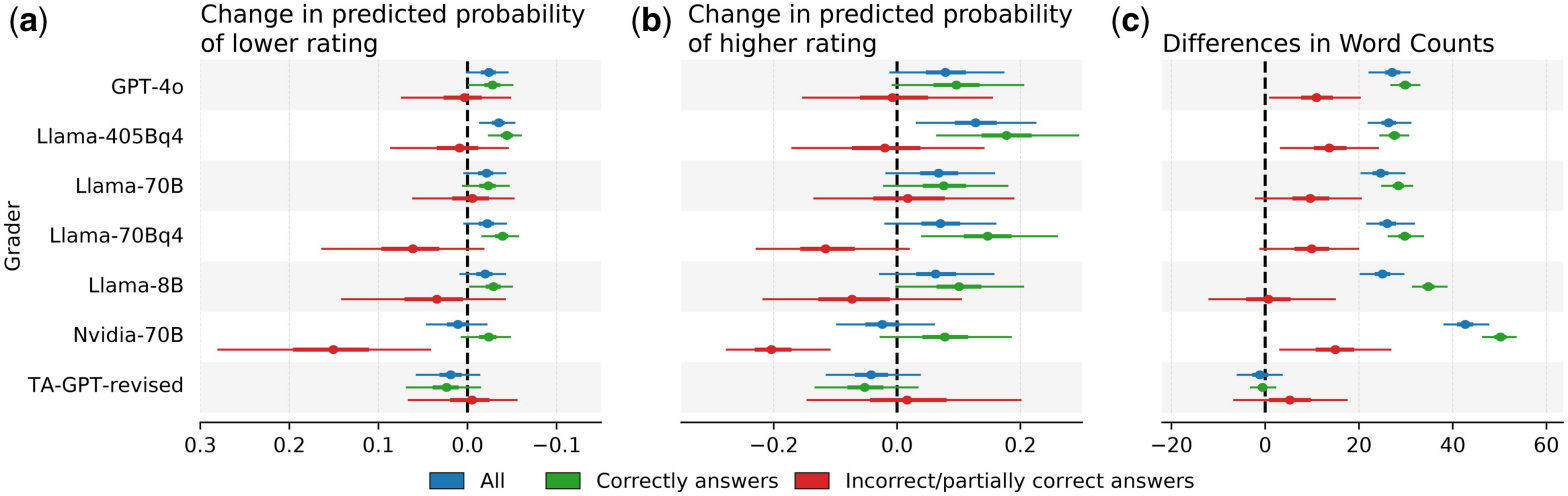
Student preferences for individual graders. Due to the correlation between group factors during HMC sampling, we use the TA group as the reference and report differences in satisfaction relative to TA-written feedback. Since grading group factors are difficult to interpret directly, panels (a) and (b) show the average change in the probability that students would assign a lower (a) or higher (b) satisfaction rating when feedback is generated by each grader compared to TA. Panel (c) shows the average differences in word counts in the generated feedback compared to TA-written feedback. 95% credible intervals (CI) in (a) and (b) are obtained using the highest density interval, while the confidence intervals in (c) are obtained using bootstrap samples.

With the exception of feedback generated by Llama-405Bq4, whose feedback students slightly preferred, Fig. 5a and b suggest no significant preference for any particular grader. However, when examining feedback preferences separately for correctly and incorrectly answered questions, a more nuanced pattern emerges. To explore this, we extend the model from Equation (1) and introduce two sets of grading group factors: one for correctly and one for incorrectly answered questions. We tie each pair of grader factors into a hierarchical model via a Gaussian hyperprior. Figure 5a shows that students generally did not rate LLM feedback lower than TA feedback. The only notable exception is Nvidia-70B, whose feedback to incorrectly answered questions led to a roughly 15% higher likelihood of negative ratings compared to TAs. This suggests that, with the exception of Nvidia-70B, LLM-generated feedback is generally on par with that of human TAs. Figure 5b shows how much more likely students would be to rate feedback higher if it were written by an LLM. Interestingly, although the changes in probabilities are relatively modest, students appear to prefer LLM-written feedback over feedback written by human TAs, particularly for correctly answered questions. For incorrect answers, satisfaction with LLM and TA feedback was comparable. One possible explanation for this discrepancy is the difference in feedback styles. When a student’s answer is correct, human TAs often provide minimal feedback, such as “ok” or “That’s right.” When the answer is incorrect or partially correct, TA feedback tends to focus on the missing or incorrect aspects of the answer and explaining the correct solution, thus producing longer feedback. Conversely, LLMs tend to provide much longer feedback both for correctly and incorrectly answered questions (see Fig. 5c).

### 4.4 Student attitudes toward LLM-based grading

**Figure 6.**
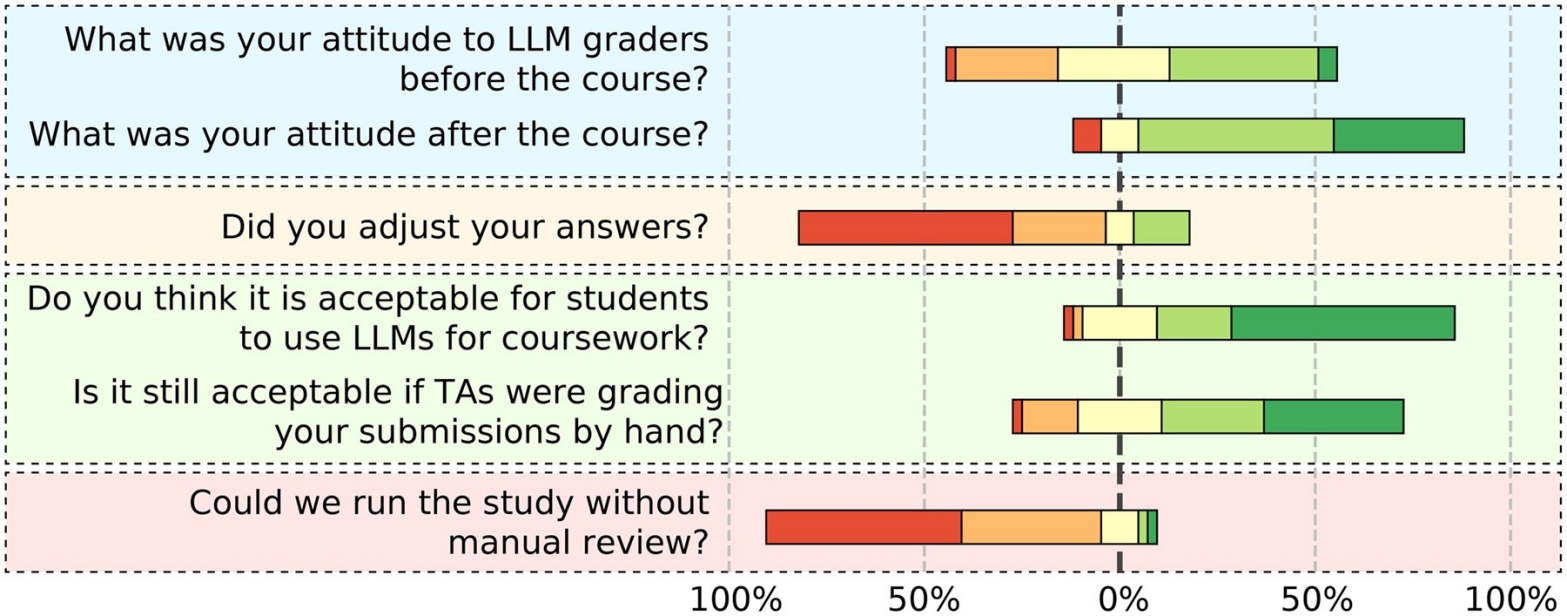
Results of the final survey. Questions are asked on a 5-point Likert scale. Red bar colors correspond to negative attitudes and disagreement, while green bar colors indicate positive attitudes and agreement.

At the end of the semester, we presented the preliminary findings of this study to the students in the classroom. Following the session, students were asked to complete a short, anonymous survey regarding their attitudes toward the use of LLMs as assignment graders and whether their attitudes had changed over the course of the semester. A total of 42 students responded to the survey. Selected results are shown in Fig. 6.

We first asked students whether they felt it was appropriate for us to grade their assignments using LLMs before the beginning of the course. Student responses were mixed, with an average score of 3.2. Encouragingly, after completing the course, students were much more open to LLM graders, with the average score increasing to over 4.0.

We also asked students whether they had used any LLM-enabled tools while working on the assignments. Over 92% of students reported using such tools, with 90% using ChatGPT for solving programming tasks and answering essay-style questions and 46% using Copilot for code generation in programming tasks. Inevitably, in some cases, this devolves into LLMs grading the output of other LLMs. While attempts have been made to detect LLM-generated content, educators will increasingly have to find ways to deal with LLM-generated content submitted as their own by students. One student also reported using ChatGPT to better understand the assignment instructions. Most students expressed that it is fair for them to use LLMs when solving the assignments if graded by LLMS (μ=4.2), but feel more hesitant about it when graded by human TAs (μ=3.8) (Fig. 6). Although students knew their answers might be graded by an LLM, they largely reported on keeping their answering style.

In the present study, students could request a manual review of their grades at any time. Consistent with prior research (Chiang *et al.* 2024), students strongly felt it would be unacceptable not to have the option to request a manual review. In practice, requests for manual reviews were rare; among the 498 total submissions, we received only three such requests (0.6%).

## 5 Recommendations and guidelines

Based on our semester-long experience and the results of our analysis, we offer the following recommendations and guidelines for incorporating LLMs into assignment grading workflows:

1. **Use structured grading rubrics:** Develop structured grading rubrics and include specific sections for explanations. This enables LLMs to provide clearer feedback, particularly for more difficult questions.
2. **Include graded examples:** Include graded examples of the student submissions. These examples help LLMs better understand TA grading style and expectations.
3. **Test new grading rubrics:** When preparing grading rubrics, conduct a dry run on a sample of manually graded student submissions to identify potential systematic grading errors. Pay close attention to the wording of criteria, as LLMs may sometimes be unpredictably pedantic, and small changes in wording can significantly impact grading accuracy. Any refinements should further be validated, ideally on a new sample of student submissions, to avoid overfitting.
4. **Open-source LLMs:** If selecting an open-source LLM, we recommend selecting the largest LLM your hardware can support. Quantization appears to have negligible effects on an LLM’s grading capabilities compared to their full-precision counterparts, so prioritize larger quantized models over smaller full-precision models. In terms of grading performance, open-source LLMs perform as well as their commercial counterparts.
5. **Allow requesting manual review:** Provide students with the option to request a manual review of their grades, as LLMs still make occasional errors.

## 6 Conclusion

We presented a study on the use of LLMs for grading written assignments in the Introduction to Bioinformatics course during the 2024–25 academic year. By implementing and evaluating LLM graders in a real-world classroom setting, we found that automated grading can achieve performance comparable to that of human TAs in both scoring and feedback generation. Our findings show that well-designed grading rubrics and examples graded by TAs help make automated grading work well in courses with many students.

Our results show that open-source models perform on par with commercial alternatives both in terms of grading accuracy and feedback satisfaction. For example, Llama-405Bq4 achieved comparable results to GPT-4o across all evaluated criteria. This suggests that, with sufficient hardware resources, universities could deploy their own instances of LLM graders without compromising performance. Such an approach could also alleviate the substantial financial costs associated with commercial solutions, as highlighted by Chiang *et al.* (2024). While the comparable performance of open-source models is promising, their high hardware demands may pose challenges for many university departments. Recent research has focused on developing smaller models that can achieve similar performance to larger ones (Team *et al.* 2024), and we envision that, in the future, grading could be performed locally on consumer-grade laptops, making it accessible to everyone. However, this capability is not yet a reality. An alternative approach could involve fine-tuning existing models to enhance performance, as studies have shown that even small amounts of domain-specific data can lead to significant improvements (Katuka *et al.* 2024).

Our study has several limitations. First, due to their probabilistic nature, LLMs can generate different grading responses even when prompted identically multiple times. Although adjusting the temperature parameter can reduce variability, some randomness typically persists (Jauhiainen and Guerra 2024). While local models produced deterministic results, GPT-4o showed minor variability in feedback when prompted identically multiple times, but its assigned grade remained consistent. Second, previous studies have reported instances of students engaging in prompt-hacking, where submissions contain deceptive instructions, such as directing the LLM to assign the maximum possible score (Chiang and Lee 2023). To mitigate this, we incorporated anticheating measures into our system prompts; however, we did not observe any prompt-hacking attempts throughout the semester. While we did not explicitly prohibit this behavior, students may have refrained from such practices, knowing that their submissions could be reviewed by human TAs. In an LLM-only grading environment, students might be more inclined to exploit such vulnerabilities. Therefore, implementing robust safeguards to detect and prevent malicious input remains essential.

Our study introduces an innovative approach to automated grading by conducting a real-classroom evaluation in the Introduction to Bioinformatics course, a carefully designed program previously reported at ISMB 2024 (Poličar *et al.* 2024). With a large number of students participating in a randomized study, we systematically compared the performance of multiple open-source and commercial LLMs. Our findings demonstrate that open-source models can achieve results comparable to commercial alternatives, offering institutions greater control over their grading processes. These contributions provide valuable insights for the broader adoption of LLM-based grading in structured homework, project reports, and exams in bioinformatics education and beyond.

Conflict of interest: No competing interest is declared.
